# Impact of food system interventions to increase fruit and vegetable intake among urban adults in Nigeria and Vietnam

**DOI:** 10.1007/s12571-025-01529-5

**Published:** 2025-04-24

**Authors:** Giulia Pastori, Elise F. Talsma, Edith J. M. Feskens, Le Thi Huong, Folake O. Samuel, Oluyemisi F. Shittu, Toluwalope E. Eyinla, Alan de Brauw, Kate Ambler, Sigrid Wertheim-Heck, Ricardo Hernandez, Brice Even, Gennifer Meldrum, Amanda De Filippo, Le Thi Thanh Xuan, Ngo Thi Ha Phuong, Truong Tuyet Mai, Mark Lundy, Inge D. Brouwer

**Affiliations:** 1 Division of Human Nutrition and Health, Wageningen University and Research, Wageningen, The Netherlands; 2 Institute for Preventive Medicine and Public Health, Hanoi Medical University, Hanoi, Vietnam; 3 Department of Human Nutrition and Dietetics, University of Ibadan, Ibadan, Nigeria; 4 The Alliance of Bioversity International and the International Center for Tropical Agriculture, Cali, Colombia; 5 International Food Policy Research Institute, Washington, DC USA; 6 National Institute of Nutrition, Hanoi, Vietnam

**Keywords:** Healthy diet, Impact evaluation, Dietary assessment, Accessibility, Acceptability, Affordability

## Abstract

Fruit and vegetable consumption is below the WHO recommendations, globally, in Southeast Asia, and in West Africa. Affordability, accessibility, and acceptability are the main drivers of consumption. Nutrition-sensitive food system interventions that address these drivers may be effective in increasing fruit and vegetable consumption. This study evaluates the effect of an integrated nutrition-sensitive program that aimed to increase fruit and vegetable consumption in low-income urban adults in Hanoi, Vietnam (*n* = 582), and Ibadan, Nigeria (*n* = 626), through the simultaneous implementation of three interventions at the market and consumer levels. Fruit and vegetable intake data were collected after eight months of exposure to the program with repeated quantitative 24-hour recalls and exposure effect was estimated with inverse probability weighting with regression adjustment, adjusting for potential confounders. The impact size on total fruits and vegetables (144 g/d, 95%CI 93, 196), fruits (137 g/d, 95%CI 93, 183) and vegetables (6 g/d, 95%CI -12, 24) showed that intake was higher in the exposed Nigerian population than the control group. In Vietnam, intakes of fruits and vegetables in the exposed group did not statistically differ from the control group when controlling for differences between groups with propensity scores. Participants exposed to all three interventions reported slightly higher intakes compared to those who were exposed to fewer interventions, but these differences were not statistically significant. Integrated approaches of nutrition-sensitive food system interventions need to be implemented to increase fruit and vegetable consumption. Co-creation of interventions provides the possibility to address the different drivers and barriers of healthy diets specific of the context.

**Supplementary Information:**

The online version contains supplementary material available at 10.1007/s12571-025-01529-5.

## Introduction

Dietary guidelines vary across countries but healthy diets are globally defined as rich in fruits and vegetables, nuts and seeds and unrefined grains, and low in highly processed foods, sugar, salt and fats (WHO, 2018). Specifically, a considerable daily intake of diverse fruits and vegetables (400 g/d) (WHO, 2003) is recommended as protective for all-cause mortality and prevent micronutrient deficiencies as well as diet-related non-communicable diseases, such as cancer, diabetes and cardiovascular diseases, due to their antioxidant compounds, vitamins, minerals and fibre content (Aune et al., 2017; Wang et al., 2014). However, globally, fruit and vegetable intake is below the recommendations with, on average, less than 100 g of daily fruits and less than 200 g of daily vegetables consumed (Afshin et al., 2019). Especially in many low- and middle-income countries (LMICs) the average intake of fruits and vegetables is lower than recommended (Frank et al., 2019; Hall et al., 2009), with Southeast Asia (100 g/d fruits; 150 g/d vegetables) and West Africa (80 g/d fruits; 110 g/d vegetables) being among the regions with the lowest consumption (Afshin et al., 2019). Therefore, promoting increased fruit and vegetable intake is one of the key strategies towards healthier diets (Harris et al., 2023).

Fruit and vegetable consumption is driven by multiple interlinked social, environmental and economic barriers and enhancers (de Brauw et al., 2019; Raaijmakers et al., 2018), both at the individual and external levels. At the individual level, affordability, accessibility and acceptability are the main drivers of consumption (Turner et al., 2018). Affordability is the combination of individual purchasing power and market prices and largely determines what food items are bought and consumed. Diet quality is positively correlated to the cost of the diet, resulting in healthy diets being unaffordable for many people, especially low-income groups (Darmon & Drewnowski, 2015; de Pee & Turowska, 2022; FAO et al., 2020; Mayén et al., 2014). Accessibility refers to factors such as time, distance, space and means of transportation that enable an individual to reach the available foods. Proximity to markets and retailers, reduced travel time and having more time for cooking positively influence diet quality and food security (Kehoe et al., 2019; Laraia et al., 2004; Mathenge et al., 2023). Acceptability refers to the cultural preferences, knowledge and attitudes towards food products and consumption habits. Socio-demographic characteristics, such as education level, have been positively linked to acceptability and diet quality (Rashid et al., 2011; Thiele et al., 2004) and food preferences and choices largely vary across countries (Kniffin et al., 2012).

Numerous entry points exist to increase fruit and vegetable consumption through a food system approach, such as intervening in food production, handling, storage and processing, food trade and marketing, consumer demand, food preparation and preferences (FAO, 2017; Huse et al., 2022). In recent years, it has been recognized that the effectiveness of food system programmes is higher when incorporating multiple strategies, implemented for a longer period and co-created with the target groups (Brouwer et al., 2021; Ciliska et al., 2000). Specifically, community-based multicomponent interventions were found to have positive effects on fruit and vegetable consumption, although studies in LMICs are limited (Pomerleau et al., 2005).

The “Fruit and vegetable intake in Vietnam and Nigeria” (FVN) is a multi-country and multi-sectorial project that aimed to improve the quality of the diet in urban and peri-urban contexts of Hanoi, Vietnam, and Ibadan, Nigeria, focusing on increasing fruit and vegetable consumption. This nutrition-sensitive project included a package of three distinct interventions that were co-created with vendors and consumers attempting to address accessibility, affordability, and acceptability.

Our primary aim was to evaluate the overall FVN project by assessing the fruit and vegetable intake of the target populations at the end of the intervention compared with the intakes of similar populations not exposed to the project, hypothesizing that the exposed groups would consume more fruits and vegetables. Secondly, as an exploratory analysis, we estimated the effect of the combination of the three interventions co-created to address accessibility, affordability and acceptability and explored whether exposure to multiple interventions could positively impact the fruit and vegetable intake more than a single intervention.

## Methods

**Fig. 1:**
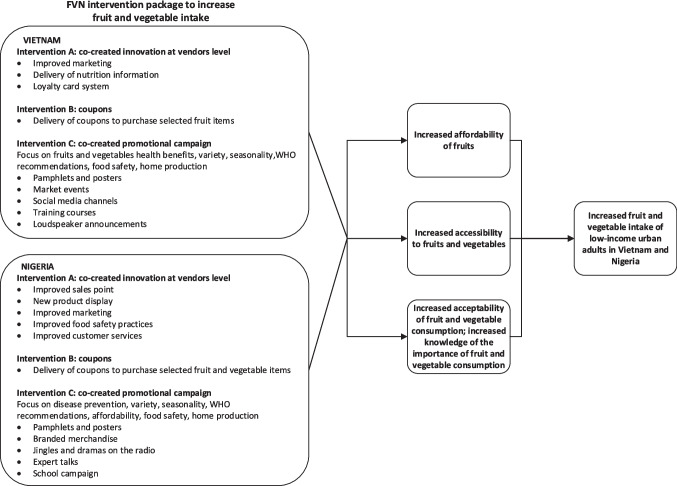
Overview of “Fruit and Vegetable intake in Vietnam and Nigeria” (FVN) program interventions and pathway for increasing fruit and vegetable intake of low-income urban Vietnamese and Nigerian study population

A cross-sectional study was conducted in Hanoi, Vietnam, and Ibadan, Nigeria, to assess the fruit and vegetable intakes after the implementation of a package of three FVN interventions among a random sample from the intervention area, compared with intakes of a random sample from a control area. The intervention package and project approach are presented in Fig. 1. Evaluations of the single FVN interventions are reported elsewhere (Even et al., 2024; De Filippo et al., 2021) and in the process of publication.

### Study population and areas

The evaluation was conducted among adults aged 20–51 years (females and males) of low-income households in urban and peri-urban neighbourhoods of two cities in Vietnam (Hanoi) and Nigeria (Ibadan). In Vietnam, the urban neighbourhoods of Đống Đa (exposed) and Nam Từ Liêm (control) were selected because of their central position and higher prevalence of low-income households; the peri-urban neighbourhoods of Hà Đông (exposed) and Thanh Trì (control) were selected as they have recently been included into the city’s boundaries and have a high prevalence of low-income households. In Nigeria, the urban neighbourhoods of Abàeja (exposed) and Apete (control) and the peri-urban neighbourhoods of Bagadajé (exposed) and Ariyibi (control) were selected based on the high prevalence of low-income households. The control neighbourhoods were selected at the end of the interventions in both countries. In Vietnam, a list of eligible households was developed for each study neighbourhood by the community health workers of each neighbourhood; while in Nigeria, this list was developed for each study neighbourhood by the local research team. In both countries, the lists of eligible households were compiled prior the start (for the exposed groups in 2019), and at the end (for the control groups in 2021) of the interventions. In Vietnam, low-income households were defined as households with a monthly income < 2,650,000 Vietnamese dong (VND) per capita based on the lowest 30% of the population in 2017 (World Bank, 2018); in Nigeria, low-income households were identified by the local research team evaluating the status of the house (Appendix Table 1). From the compiled lists in both countries, a total of 100 households including a non-pregnant and non-lactating female were randomly selected from each neighbourhood and checked for eligibility. For the intervention neighbourhoods females of reproductive age (18–49) were selected at the start of the intervention period, being aged 20–51 years after the two-year period of preparation and implementation of the interventions. For the control neighbourhoods, females aged 20–51 years were selected at the end of the interventions, to align with the age of the study population of the intervention neighbourhoods. In both the intervention and control groups a male from the same household was included only if present in the household at the time of the interview using convenience sampling. The selection of the population is represented in the supplementary material (Appendix Fig. 1). The dropouts at the end of the interventions occurred because the respondents relocated to a different area, were not available to be interviewed or were not reached during the data collection.

### Ethical approval

Ethical approvals were obtained before the start of the FVN project from the Hanoi Medical University Institutional Review Board in Hanoi (45–18/HMU-IRB), the University of Ibadan/University College Hospital Ethical Review Committee (UI/UCH-ERC) in Nigeria (HNHREC/05/01/2008a), and the International Food Policy Research Institute’s Institutional Review Board (IFPRI IRB-007490). The randomized control trial associated with the affordability intervention was registered with the American Economic Association’s registry (AEARCTR-0007701). All participants signed an informed consent before the start of the study.

### FVN interventions

The FVN project consisted of a package of three interventions aiming to increase fruit and vegetable intake of low-income urban and peri-urban consumers intervening at both the market and demand sides (Fig. 1). It included interventions aiming to address accessibility, affordability, and acceptability of fruits and vegetables. The three country-specific interventions were designed based on preliminary information on fruit and vegetable consumption and practices of the study population (Pastori et al., 2020a), on a market (Hernandez et al., 2019) and a seasonality assessment (Bioversity International & NIN, 2019; Brouwer et al., 2019) of the study areas, and analysis of the main barriers for fruit and vegetable consumption (De Filippo et al., 2021). The first intervention (A) was designed using a participatory co-creation approach with local fruit and vegetable vendors and aimed to address accessibility of fruits and vegetables. This intervention included various innovations such as improved point of sales and product display (Nigeria), improved marketing (Vietnam and Nigeria), delivery of nutrition information to consumers (Vietnam and Nigeria), improved food safety and customer service practices (Nigeria) and set-up of a loyalty card system (Vietnam). This intervention was implemented for eight months in both countries. The second intervention (B) addressed affordability and consisted of the distribution of coupons of two different monetary values (low: 30,000 VND and 400 Nigerian naira (₦); high: 60,000 VND and 800 ₦) to purchase a selection of items (8 fruits in Vietnam; 7 fruits and 2 vegetables in Nigeria) from selected fruit and vegetable vendors. Mainly fruits were selected based on a previous study showing that the affordability of fruits in Vietnam and of fruits and vegetables in Nigeria was one of the main barriers to consumption faced by our study populations (De Filippo et al., 2021). Coupons were delivered to randomly selected sample households, expired two weeks after they were received and could be redeemed at a set of participating vendors at selected markets. In Vietnam, coupons were delivered on a biweekly basis, by a delivery service for the first two months and then by community health workers. In Nigeria, coupons were delivered by project staff every week. This intervention was implemented for five months in both countries. The third intervention (C), addressing acceptability and developed and reviewed through four co-creation workshops engaging low-income consumers, involved a series of neighbourhood-specific campaigns aimed at promoting the importance of adequate daily fruit and vegetable consumption. In Vietnam, communication materials (pamphlets, posters) focused messaging around fruit and vegetable health benefits, variety, seasonality, recommended intake, food safety and home production. These were disseminated by local health centres through social media platforms, market events, training courses, and, only in Hà Đông, by loudspeaker announcements. In Nigeria, messages in the communication materials (pamphlets, posters, branded merchandise, jingles, dramas, and expert talks) highlighted fruit and vegetable disease prevention, affordability, food safety, home production, variety and seasonality, and campaigns were carried out through radio stations, public health centres, religious centres and schools. This intervention was implemented for eight months in both countries. In both countries, interventions A and C started at the same time (December 2020 in Vietnam; February 2021 in Nigeria), intervention B started three months later (February 2021 in Vietnam; June 2021 in Nigeria), and all interventions ended at the same time (July 2021 in Vietnam; October 2021 in Nigeria). All three interventions targeted consumers and fruit and vegetable vendors within the selected study neighbourhoods. Therefore, all respondents could have been potentially exposed to the intervention at the vendor level and the promotional campaigns. In contrast, the coupon system followed a randomized control trial design with part of the respondents receiving the intervention (coupons) and others not (control group).

### Dietary and exposure assessment

In both countries, a repeated quantitative multi-pass 24-hour recall (24hR) (Gibson & Ferguson, 2008) was administered by trained local interviewers to obtain information on food consumption. For each respondent in both countries and study areas, two non-consecutive 24hRs were collected with at least two days and at most one month in between interviews, to avoid dependency of intakes and seasonal effect. Description and quantities of all foods and drinks consumed by the participant in and outside of the home in the previous 24 hours were recorded. Interviewers also recorded the time of day of the eating episode, the place of preparation, the frequency of consumption and the place of purchase of each ingredient. Additionally, a questionnaire was administrated using KoboToolbox (KoboToolbox Inc., 2023) software to obtain information on the exposure to the interventions. Information on the exposure to interventions A and C was self-reported by respondents, while exposure to intervention B was based on the random allocation to the coupon system. Finally, the respondent’s weight and height were measured twice using a scale (Tanita in Vietnam; SECA 813 in Nigeria) and stadiometer (SECA 213 in both countries) following standard methods (Cashin & Oot, 2018). From the mean of the two measurements, body mass index (BMI) was calculated as indicators of underweight (BMI < 18.5 kg/m^2^), normal range (BMI 18–24.99 kg/m^2^) and overweight/obesity (BMI ≥ 25 kg/m^2^) (Cashin & Oot, 2018). The FVN project preparation started in 2019 and some delays occurred due to the COVID-19 pandemic. Data were collected between October-December 2021 in Vietnam and November–December 2021 in Nigeria. In Vietnam, data collection took place about three months after the end of the implementation of the FVN interventions because of restrictions on home visits imposed by the government due to the COVID-19 pandemic. In Nigeria data was collected immediately after the end of the FVN interventions.

### Data processing and analysis

Data from the 24hR interviews were checked for missing values, inconsistencies, and extreme high and low intakes. From these, information on fruit and vegetable intakes was extracted for the aim of this study. From each recall, daily fruit and vegetable intakes were calculated by multiplying the quantity recorded in each 24hR by the weekly frequency of consumption and divided by seven days. Then the average of the two recalls was calculated and used for analysis. For each country, descriptive statistics were used to explore the sociodemographic characteristics of the exposed and control groups.

Inverse probability weighting with regression adjustment was used to compare intakes of total fruits and vegetables, and fruits and vegetables separately, between the exposure group and the control group. The estimation procedure first estimated propensity scores, using a list including age, sex, neighbourhood, BMI, household size, education, and occupation as potential predictors. The predictors for each country were determined from that list using a LASSO procedure.^1^The resulting propensity scores were then used as regression weights in a second step (Glynn & Quinn, 2009), again controlling for the predictors in the second stage, completing the regression adjustment. The analysis performed can be considered an intent-to-treat analysis, as all respondents from the intervention areas are considered as exposed to the interventions. Generalized mixed models were used to explore the impact of the degree of exposure on total fruits and vegetables, and fruits and vegetables separately. Additionally, trend analysis was performed to explore the hypothesis of an ordered increase of these outcomes across the degree of exposure to the different interventions in the exposed groups. As the study populations included females and males living in the same households a random effect for household was included in the mixed model. Bootstrapping was applied for standard error estimation as some outcomes were skewed.

All models were adjusted for age, sex, neighbourhood, BMI, household size, education, and occupation, variables selected for their possible effect on the amount of fruit and vegetable consumed. Furthermore, fruit intake analyses were repeated after ranking fruit intakes instead of using actual amounts consumed due to the skewness of the distribution towards zero consumption for the large number of non-consumers in the study population. Sensitivity analysis was also performed to check the contribution of outliers for high intakes (> 95% of the distribution). Since no differences were found with and without ranking, and with and without outliers, all observed actual amount consumed and data points were included in the final analyses. Effect modification by sex was investigated by adding an interaction term to the models. We performed all analyses in SPSS Statistics (version 28.0.1.1) (IBM Corp., 2021).

## Results

In Vietnam, the exposed group (*n* = 283) included more females (70%) compared to the control group (*n* = 299; 60% females) and the average age was 38 years for both groups (Table 1). Most of the exposed respondents were skilled employed (38%), while the majority of the control group was unskilled (45%). Although education level differed between the groups, more than half of the respondents finished high school in both groups (60% exposed; 62% control) and the majority had a normal BMI (80% exposed; 86% control). Among the exposed group, 54% of the respondents reported having received loyalty cards or nutrition advice from the fruit and vegetable vendors (intervention A); 72% received the coupons to purchase fruits (intervention B); and only 31% reported being exposed to the promotional campaigns on the importance of daily fruit and vegetable consumption (intervention C). Overall, among the study participants in the exposed group, 14%, 34% and 29% were exposed to one, two and three interventions respectively and 22% did not perceived to be exposed to interventions at all.

**Table 1:** General characteristics of the Vietnamese and Nigerian study population by exposure to the interventions of the FVN project

	Vietnam			Nigeria		
	Exposed (*n* = 283)	Control (*n* = 299)	*p*-value	Exposed (*n* = 291)	Control (*n* = 335)	*p*-value
**Female, %**	69.6	60.2	0.013	67.7	64.2	0.273
**Urban, %**	53.7	53.2	0.931	47.4	49.0	0.679
**Age, mean (sd)**	37.9 (7.2)	37.6 (7.3)	0.956	38.1 (8.1)	37.3 (7.7)	0.360
**Household size, mean (sd)**	4.9 (1.3)	4.3 (1.2)	0.240	5.6 (2.0)	5.2 (1.6)	< 0.001
**Occupation, %**			< 0.001			< 0.001
Skilled employment	38.5	38.8		16.8	19.1	
Unskilled employment	31.1	45.2		3.4	6.9	
Own business	20.5	9.0		64.6	53.7	
Unemployed	9.9	7.0		15.1	20.3	
**Education, %**			< 0.001			< 0.001
No education	1.1	0		4.5	3.6	
Primary school	7.8	2.3		27.8	19.4	
Secondary school	17.0	20.4		41.2	51.6	
High school	60.4	61.9		22.7	26.9	
Above high school	13.8	15.4		3.8	1.8	
**BMI, mean (sd)**	21.8 (2.7)	22.0 (2.5)	0.335	24.6 (5.2)	24.7 (5.5)	0.702
Underweight, %	8.0	4.7		7.1	3.9	
Overweight/obesity, %	11.5	9.6		38.6	39.0	
**Fruit and vegetable intake > 400 g/d, %**	56.6	58.8	0.596	49.2	25.3	< 0.001
**Fruit and vegetable intake, g/d**						
Mean (sd)	451 (167)	442 (139)		445 (311)	297 (222)	
Median (iqr)	421 (208)	430 (165)		390 (419)	238 (272)	
**Fruit intake, g/d**						
Mean (sd)	167 (126)	145 (99)		283 (269)	143 (184)	
Median (iqr)	145 (135)	129 (113)		230 (372)	80 (215)	
**Vegetable intake, g/d**						
Mean (sd)	284 (108)	296 (106)		162 (107)	154 (103)	
Median (iqr)	268 (148)	285 (134)		133 (119)	130 (107)	
**Exposure, %**						
Intervention A	54.0			92.4		
Intervention B	72.0			87.6		
Intervention C	31.0			69.1		
1 intervention	14.3			8.1		
2 interventions	34.3			34.2		
3 interventions	29.0			57.6		
Not exposed	22.4			0		

p−value, exposed compared to control; sd=standard deviation; BMI=Body Mass Index; Underweight: BMI <18.5; Normal range: BMI 18.5 – 24.99; Overweight/obesity: BMI≥25; g/d=grams per day; iqr=interquartile range; all exposure are self−reported except to intervention B based on random allocation; A=intervention at vendor level; B=coupon system intervention; C=promotional campaign intervention; 1=exposed to one intervention, 2=exposed to two interventions; 3=exposed to 3 interventions

In Nigeria, the exposed (*n* = 291) and the control group (*n* = 335) were comparable regarding the proportion of females, participants from urban areas (68% and 64% females, 47% and 49% urban), average age (38 ± 8.1 years exposed; 37 ± 7.7 years control) and in both groups 39% were overweight/obese (Table 1). Respondents in the exposed group lived in larger households, composed on average of 6 ± 1.7 members, compared to respondents in the control group (5 ± 1.7 members). Although they differed in occupation and education, a large proportion of the respondents in both groups ran their own business (65% exposed; 54% control) and finished secondary school (41% exposed; 52% control). Among the study participant in the exposed group, almost all respondents (92%) reported having noticed the new display, aprons and umbrellas or the improved hygienic practices of the fruit and vegetable vendors (intervention A); or have received the coupons to purchase fruit and vegetable (88%) (intervention B); and 70% were exposed to at least one of the activities implemented to increase awareness on the importance of daily fruit and vegetable consumption (intervention C). All respondents in the exposed group reported having been exposed to at least one intervention, more than half were exposed to all three interventions (58%), one-third to two interventions (34%) and only 8% of the respondents to one intervention.

### Comparison between exposed and control group.

More than half of the Vietnamese respondents met the daily global recommendations for fruit and vegetable intake (57% exposed; 59% control) with an average intake of 451 g/d and 442 g/d, respectively (Table 1). The results with the matching variables are reported in the supplementary material (Appendix Table 2). After matching, the two groups had comparable total daily fruit and vegetable intake (difference (exposure minus control) 5.2 g/d, 95%CI −22, 33) (Table 2). The exposed group had comparable intake of fruits (difference 13.0 g/d, 95%CI −7, 32), and vegetables relative to the control group (difference −7.8 g/d, 95%CI −26, 10). No significant interaction between sex and exposure to the interventions was found (Appendix Table 3). However, for fruit intake stratified analysis showed a difference of 21.4 g/d (95%CI 1, 46) for females and 14.2 (95%CI −14, 44) for males, compared to the control group (Appendix Table 4).

**Table 2:** Average treatment effects on fruit and vegetable consumption (g/d) for Vietnamese and for Nigerian study population

	Vietnam (*n* = 582)					Nigeria (*n* = 626)				
	*Effect size*	*SE*	*95% CI*		*p*	*Effect size*	*SE*	*95% CI*		*p*
Fruits and vegetables	5.2	14.0	−22.3	32.8	0.710	144.2	26.1	93.0	195.5	< 0.001
Fruits	13.0	10.0	−6.5	32.6	0.191	138.0	22.7	93.5	182.5	< 0.001
Vegetables	−7.8	9.3	−26.0	10.4	0.398	6.5	9.2	−11.8	24.3	0.496

Control group set as reference. Groups matched for age, sex, area, education, occupation, BMI, household size; SE=standard error; CI=confidence interval; SE and p−values from bootstrapping

In Nigeria, a higher proportion of exposed respondents met the daily recommendation for fruit and vegetable consumption compared to the control group (49% exposed; 25% control) (Table 1) with an average intake of 445 g/d and 297 g/d, respectively. The exposed group consumed considerably higher amounts of total fruits and vegetables (difference 144 g/d, 95%CI 93, 196) compared to the control group (Table 2). The results with the matching variables are reported in the supplementary material (Appendix Table 5). The difference was mainly due to higher fruit intake (difference 138 g/d, 95%CI 94, 183); but a small difference was found also in vegetable intake between groups (difference 6.5 g/d, 95%CI −12, 24). No evidence of effect modification by sex was observed (Appendix Table 6).

### Fruit and vegetable consumption across degree of intervention exposure

We did not find any strong association between the degree of intervention exposure and fruit and (Table 3). Compared to reporting being exposed to 3 interventions, Vietnamese respondents not exposed to any intervention or exposed to 1 or 2 intervention(s) had only slightly lower intakes of total fruits and vegetables, or fruit intake and vegetable intake separately, with all confidence intervals including the null. The *p*-values for linear trend suggests that there is no evidence of a dose–response relationship between the amount of intervention exposure and intakes of fruits and/or vegetables. Nigerian respondents exposed to 1 or 2 interventions had relatively lower intakes compared to respondents exposed to all 3 interventions (Table 3). The largest differences from the maximum exposure were found in the group with the lowest degree of exposure for total fruit and vegetable (difference −127 g/d, 95%CI −370, 280) and fruit (difference −117 g/d, 95%CI −272, 81) intakes; the accompanying *p*-values for linear trend suggest a trend for fruit intake across the exposure groups (*p* = 0.08).

**Table 3:** Average treatment effects on fruit and vegetable consumption (g/d) across exposure to interventions in the Vietnamese and the Nigerian study population, exposed to 3 interventions (maximum) as reference

	**Viet**n**am** (*n* = 582)					Nigeria (*n* = 626)				
	*Effect size*	*SE*	*95% CI*		*p*	*Effect size*	*SE*	*95% CI*		*p*
Fruit and vegetable										
Not Exposed	−44.6	39.7	−127.7	47.3	0.333	NA				
Exposed to 1 intervention	−22.9	31.1	−94.0	43.8	0.568	−127.2	84.4	−370.4	280.5	0.197
Exposed to 2 interventions	−28.3	31.1	−98.8	60.1	0.453	−47.6	59.8	−155.3	37.9	0.408
p trend					0.459					0.146
Fruit										
Not Exposed	−34.1	33.4	−103.0	30.5	0.356	NA				
Exposed to 1 intervention	−24.0	21.9	−66.2	18.1	0.377	−117.9	70.3	−271.9	80.8	0.114
Exposed to 2 interventions	−26.5	22.3	−87.6	60.2	0.395	−42.2	50.2	−127.5	10.7	0.375
p trend					0.358					0.082
Vegetable										
Not Exposed	−6.5	30.5	−79.8	67.0	0.868	NA				
Exposed to 1 intervention	1.6	18.8	−44.1	42.4	0.946	−19.8	23.1	−271.9	80.8	0.688
Exposed to 2 interventions	−7.2	16.8	−42.0	32.8	0.737	−4.2	16.2	−39.8	43.6	0.782
p trend					0.986					0.655

Mixed model adjusted for age, sex, area, education, occupation, BMI, household size. Household included as random factor, and bootstrap applied; SE=standard error; CI=confidence interval; SE and p−values from bootstrapping; NA=not applicable, all Nigerian respondents reported to be exposed to at least one intervention

## Discussion

This study evaluated the potential impact of an integrated nutrition-sensitive program on fruit and vegetable consumption among low-income adults in urban and peri-urban areas in Hanoi, Vietnam, and Ibadan, Nigeria. In Nigeria, subjects living in the intervention areas had higher total fruit and vegetable intake compared to a group from the control area. The Nigerian exposed group consumed particularly more fruits compared to the control group rather than vegetables. In Vietnam, the exposed group consumed slightly more fruits and vegetables, but the difference was not large. Moreover, in our additional exploratory analysis, we could not determine a clear dose–response relationship of exposure to multiple interventions since we found a slight but not significant positive trend on fruit and/or vegetable intake in Nigeria but not in Vietnam. The differences of results found in the two countries highlights the relevance of context-specific approaches and co-creation of the interventions that aim to improve diet quality.

Higher fruit consumption among subjects from the intervention areas was, as expected, consistent with our study hypothesis. In Nigeria, the effect on fruit consumption was nutritionally meaningful, as the exposed groups reached the recommended amount for a healthy diet, largely different compared to the control group. The coupon intervention, in both countries, and the intervention at vendor level in Vietnam, addressed accessibility and affordability of mainly fruits rather than vegetables. In line with other studies, lack of financial means is one of the main drivers of low fruit and vegetable consumption (Miller et al., 2016), unhealthy diets and food choices in poor-resource settings (FAO et al., 2022; Headey et al., 2023; Mayén et al., 2014). On the other hand, affordability seemed not to be an issue for vegetable consumption in our Vietnamese setting, contrary to Nigeria. A possible explanation is that seasonal vegetables are inexpensive even for low-income populations in Vietnam, hence cheap vegetables are affordable all year round. In addition, in general, price-elasticity is lower for vegetables compared to fruits (Wertheim-Heck et al., 2015) and purchase of vegetables is less prone to price fluctuations.

The provision of food vouchers to purchase specific nutritious food items is an effective social protection intervention at food system level aiming to improve diet quality and nutritional status (Devereux & Nzabamwita, 2018). It provides a dual effect compared to cash transfers: a direct price discount on the specific food item; and indirect information and advertisement of the product (Aker, 2013; Dong & Leibtag, 2010). This seems to be effective for the Nigerian population, highlighting the barrier of financial means to purchase fruits, the desirability of consumption but the limit on the scalability and sustainability of voucher provision in long-term. Therefore, controlling food prices at the market level and designing social assistance policies tailored for urban contexts may support a sustained access, affordability and subsequently higher intake of fruits and vegetables for our Nigerian study low-income urban populations (Cuesta et al., 2020).

In Vietnam we found a weak indication of gender differences, with a larger difference between exposed and not-exposed areas in females than in males. This may be supported by results from a focus group discussion with the study population where fruits were reported to be perceived by men as “women’s food”. However, the difference between females and males was small, and need to be explored more extensively in future studies. This especially considering that the convenience selection of men used in our study could have inflated the intake of healthy foods from men, with the exclusion of single men and less wealthy, as out for work.

Our data on dietary intakes were collected after the end of the FVN interventions, hence solely a lasting effect on the study population could have been measured. In particular, the small differences between groups found in Vietnam could have been diluted, because dietary intakes were assessed three months after the end of the program. Moreover, the period of intervention implementation was characterized by instability at market level and restrictions related to shopping practices due to the COVID-19 pandemic. This have disrupted the exposure to the interventions implemented at the markets and the purchase of fresh products due to the restricted mobility. This was the case in Vietnam, where dietary intake data were collected immediately after the end of a strict lockdown. Hence, the observed differences between the exposed areas and control may have been reduced.

In addition, the lack of difference in vegetable consumption across the groups in both countries may be due to an inherent difficulty in increasing the amount of vegetable consumption. Although consumed in low amounts, most of our study participants consume vegetables regularly (99% in Vietnam; 95% in Nigeria), in contrast to fruits, which were consumed by a lower percentage of the population (70% in Vietnam; 45% in Nigeria). Vegetables are part of the traditional diets and are consumed in meals and dishes in combination with other foods. Increasing vegetable consumption might also require increasing intake of these other foods in the dishes. We did not observe such an increase, as additional analyses showed that the consumption of all other food groups was similar between exposed and control groups. On the contrary, fruits are often consumed in between meals or as snacks, and as such the intake of fruits could be increased independent of other foods consumed. Therefore, strategies that investigate behaviour related to the acceptability, culture and desirability of larger amounts of vegetables can help in identifying entry points at the population level to promote changes in recipes and increase vegetable consumption.

In general, out-of-home consumption practices need to be taken into account, considering the common habit of purchasing meals outside in the study settings (Pastori et al., 2020a), and since this could limit the possibility to increase the amount of vegetable included in the recipes. A previous study conducted in Ibadan, reported an increased rate of urban out-of-home consumption in the last decades, and includes a variety of traditional, processed and unprocessed foods (Adeosun et al., 2022a), and it is driven by mobility practices and distance to work (Adeosun et al., 2022b). Changing recipes, may be difficult but the introduction of new recipes were found effective in increasing vegetable consumption in Nigeria (Lion et al., 2018). This is also confirmed by the reported desire of buying more fruits, expressed by our study populations during the assessment conducted before the development of the interventions (Pastori et al., 2020b, 2020c).

Our additional dose–response explorative analyses suggest that fruit and vegetable intake of the exposed population might be higher when exposed to multiple interventions compared to fewer interventions, especially in Nigeria. Previous studies support the importance of a combination of multi-sectorial and integrated approaches to increase diet quality (Barrett et al., 2020) and fruit and vegetable consumption (Hodder et al., 2019), since several factors affect the diets and consumer choices. Our food system approach targeting different agents (consumers and vendors) allowed us to develop a multi-sectorial program. The three distinct interventions were interrelated and connected and addressed drivers of consumption at the same time with different approaches to complement and reinforce each other. However, attributing the effect on a single intervention or the combination of interventions is difficult because, except for the vouchers, the other interventions were taking place in the same markets and were not possible to control due to the wide-spread nature of the internation. Therefore, the study design does not allow for robust analysis for the evaluation of possible combinations of interventions. Exposure was self-reported as it was impossible to assess the actual exposure to the interventions among fruit and vegetable vendors and to the promotional campaign. Respondents could have been unaware of being exposed to the nudges at vendors or market events which leads to an underestimation of reported exposure (Vecchio & Cavallo, 2019) as well as possible spillover of the promotional campaign in the control group. The latter, however, was not considered enough to influence our primary outcome. The use of mixed methods to further investigate the perception and experience of the respondents with qualitative assessment methods can complement the evaluation of the project. Qualitative data can be used to triangulate and explain the changes in consumption diving into the drivers of behaviours. This is relevant for public health interventions where it is difficult to control all the factors affecting fruit and vegetable consumption.

We used a quasi-observational design to evaluate the FVN project. The study design does not allow us to attribute the found effect on fruit and vegetable consumption solely to the interventions. Respondents in the exposed and control areas could have differed in baseline fruit and vegetable intakes, and the differences in education and occupation in both countries could be an indicator of difference in socioeconomic status between the groups. However, we adjusted for these covariates in our analyses and hence confounding by these factors was minimised. Furthermore, we did not account for potential differences in food environment between the neighbourhoods, hence residual confounding cannot be excluded. Regarding the implementation of the interventions it should be noted that social limitations imposed to limit the spread of COVID-19 pandemic could have affected availability of and accessibility to fresh foods, which we were not able to anticipate and control for. Lastly, eight months of intervention implementation might not have been sufficient to have an impact in the study settings, as prolonged exposures to multisectoral nutrition-sensitive interventions are shown to be more successful (Ciliska et al., 2000), and allow to control for the seasonal effect on food availability, accessibility and affordability, especially for seasonal foods such as fruit and vegetables.

## Conclusion

The multi-sectorial approach targeting multiple food system actors and addressing multiple drivers of healthy diets at the same time demonstrated a positive impact in promoting higher fruit and vegetable consumption. However, the extent of this effect was limited by specific contextual constraints, including challenges unique to each country and the unpredictable disruptions caused by the COVID-19 pandemic. Identifying and overcoming these limitations could enhance the effectiveness of the interventions. The differences between Vietnam and Nigeria highlighted the critical importance of context sensitivity and local co-creation approaches were demonstrated valuable. More extensive tinkering with real-world contextual conditions could further enhance the effectiveness of the interventions.

## Supplementary Information

Below is the link to the electronic supplementary material.

Supplementary file1 (DOCX 54 KB)

## Data Availability

The data that support the findings of this study are available on request from Inge Brouwer via the Division of Human Nutrition and Health at Wageningen University and Research.

## Footnotes

1 weighting estimator with regression adjustment has the property of being doubly robust; it estimates one model to predict treatment status, and another model to predict outcomes. The doubly robust property means that only one of the two models must be correctly specified for the model to be unbiased. The LASSO procedure is a “least absolute shrinkage and selection operator”; succinctly, it provides a method of selecting variables for inclusion by including a penalty function in regression analysis as variables are sequentially added to analysis, and selects for the optimal set of variables using that penalty function.
