# Supplementary material for: Impact of food system interventions to increase fruit and vegetable intake among urban adults in Nigeria and Vietnam
Source: Food Secur. 2025 Apr 24;17(3):641–55. doi: 10.1007/s12571-025-01529-5 (PMC12106592; doi:10.1007/s12571-025-01529-5)
Supplement: Supplementary file 1 — Supplementary file1 (DOCX 54 KB) [file 12571_2025_1529_MOESM1_ESM.docx]

**Appendix**

**Table 1** Building selection criteria for Ibadan, Nigeria

|  | **Low income** | **Non-low income** |
| --- | --- | --- |
| **Type of roof material** | corrugated | non-corrugated |
| **Type of fence** | partially fenced  no fence | fully fenced |
| **Wall plaster** | partially plastered  no plaster | fully plastered |

**Fig. S1** Flow chart of population selection

**Table 2** Comparison of fruit and vegetable consumption (g/d) between exposed and control groups with variables selected by the LASSO for Vietnam

|  | *Estimate* | | *SE* |  | *95% CI* | | *p* |
| --- | --- | --- | --- | --- | --- | --- | --- |
| **Fruits and vegetables** | | 5.2 | 14.0 |  | -22.3 | 32.8 | .710 |
| **Sex** | | -11.8 | 14.8 |  | -40.2 | 16.6 | .415 |
| **Urban** | | -39.4 | 14.6 |  | -67.2 | -10.8 | .007 |
| **Age** | | 12.1 | 10.7 |  | -8.9 | 33.1 | .260 |
| **Household size** | | 0.3 | 15.6 |  | -30.4 | 31.0 | .984 |
| **Occupation** | | -9.0 | 3.4 |  | -15.6 | -2.3 | .008 |
| **Education** | | -1.8 | 2.7 |  | -7.1 | 3.4 | .495 |
|  | |  |  |  |  |  |  |
| **Fruits** | | 13.0 | 10.0 |  | -6.5 | 32.6 | .191 |
| **Sex** | | -25.8 | 10.2 |  | -45.8 | -5.7 | .012 |
| **Urban** | | -38.9 | 10.5 |  | -59.5 | -18.3 | .000 |
| **Age** | | 3.1 | 8.3 |  | -13.2 | 19.4 | .710 |
| **Household size** | | 1.5 | 11.9 |  | -21.8 | 24.8 | .898 |
| **Occupation** | | -4.8 | 2.5 |  | -9.8 | 0.19 | .059 |
| **Education** | | -0.9 | 1.9 |  | -4.6 | 2.9 | .648 |
|  | |  |  |  |  |  |  |
| **Vegetables** | | -7.8 | 9.3 |  | -26.0 | 10.4 | .398 |
| **Sex** | | 13.9 | 10.1 |  | -6.0 | 33.8 | .169 |
| **Urban** | | -0.4 | 9.6 |  | -19.2 | 18.3 | .963 |
| **Age** | | 9.0 | 6.7 |  | -4.1 | 22.1 | .178 |
| **Household size** | | -1.2 | 10.1 |  | -21.0 | 18.6 | .906 |
| **Occupation** | | -4.2 | 2.1 |  | -8.2 | -0.1 | .044 |
| **Education** | | -1.0 | 1.7 |  | -4.4 | 2.5 | .583 |

**Table 3** Difference in intake of total fruits and vegetables, and fruits and vegetables separately between exposed and control group by gender in the Vietnamese population.

|  | Vietnam  (n = 582) | | | | |
| --- | --- | --- | --- | --- | --- |
|  | *Estimate* | *SE* | *95%CI* | | *p* |
| Fruits and vegetables |  |  |  |  |  |
| Exposed | 2.9 | 20.5 | -38.2 | 43.7 | .882 |
| Females | 4.8 | 16.7 | -27.2 | 35.5 | .770 |
| Exposed * Females | 11.7 | 27.2 | -43.1 | 66.0 | .668 |
| Fruits |  |  |  |  |  |
| Exposed | 14.2 | 14.6 | -12.6 | 44.5 | .336 |
| Females | 19.8 | 11.7 | -3.6 | 43.6 | .087 |
| Exposed * Females | 7.1 | 19.0 | -32.2 | 43.6 | .701 |
| Vegetables |  |  |  |  |  |
| Exposed | -11.4 | 15.6 | -44.2 | 18.9 | .473 |
| Females | -15.0 | 12.6 | -41.1 | 9.7 | .239 |
| Exposed * Females | 4.6 | 19.5 | -33.2 | 43.5 | .816 |

^Control group and males set as reference. Mixed model adjusted for age, area, education, occupation, BMI, household size; SE = standard error; CI = confidence interval^

**Table 4** Difference in intake of fruits between exposed and control group stratified by sex in the Vietnamese population

|  | Vietnam  (n = 582) | | | | |
| --- | --- | --- | --- | --- | --- |
|  | *Estimate* | *SE* | *95%CI* | | *p* |
| Females |  |  |  |  |  |
| Exposed | 21.4 | 12.5 | -1.0 | 46.1 | .088 |
| Males |  |  |  |  |  |
| Exposed | 14.2 | 14.8 | -13.8 | 44.4 | .341 |

^Control group and males set as reference. Mixed model adjusted for age, area, education, occupation, BMI, household size; SE = standard error; CI = confidence interval^

**Table 5** Comparison of fruit and vegetable consumption (g/d) between exposed and control groups with variables selected by the LASSO for Nigeria

|  | **Nigeria**  (n=626) | | | | |  |
| --- | --- | --- | --- | --- | --- | --- |
|  | *Estimate* | *SE* |  | *95% CI* | | *p* |
| **Fruits and vegetables** | 144.2 | 26.1 |  | 93.0 | 195.5 | <.001 |
| **Household size** | 13.3 | 26.6 |  | -38.9 | 65.6 | .616 |
| **Occupation** | -8.1 | 3.4 |  | -14.8 | -1.4 | .018 |
| **Education** | -3.4 | 4.7 |  | -12.6 | 5.9 | .476 |
|  |  |  |  |  |  |  |
| **Fruits** | 138.0 | 22.7 |  | 93.5 | 182.5 | <.001 |
| **Household size** | -0.7 | 23.1 |  | -46.0 | 44.6 | .977 |
| **Occupation** | -7.4 | 2.9 |  | -13.1 | -1.6 | .013 |
| **Education** | -3.0 | 4.3 |  | -11.4 | 5.5 | .492 |
|  |  |  |  |  |  |  |
| **Vegetables** | 6.5 | 9.2 |  | -11.8 | 24.3 | .496 |
| **Household size** | 14.0 | 9.3 |  | -4.3 | 32.3 | .134 |
| **Occupation** | -0.8 | 1.2 |  | -3.1 | 1.6 | .536 |
| **Education** | -0.4 | 1.4 |  | -3.2 | 2.4 | .775 |

**Table 6** Difference in intake of total fruits and vegetables, and fruits and vegetables separately between exposed and control group by sex in the Nigerian population

|  | Nigeria  (n = 626) | | | | |
| --- | --- | --- | --- | --- | --- |
|  | *Estimate* | *SE* | *95%CI* | | *p* |
| Fruits and vegetables |  |  |  |  |  |
| Exposed | 124.3 | 44.0 | 43.0 | 205.2 | .004 |
| Females | -3.2 | 28.5 | -60.2 | 49.5 | .908 |
| Exposed * Females | 60.4 | 52.3 | -45.2 | 168.6 | .248 |
| Fruits |  |  |  |  |  |
| Exposed | 120.6 | 36.8 | 53.4 | 189.4 | <.001 |
| Females | 9.1 | 24.3 | -44.5 | 56.8 | .734 |
| Exposed * Females | 48.9 | 45.2 | -39.4 | 135.1 | .292 |
| Vegetables |  |  |  |  |  |
| Exposed | 3.7 | 15.8 | -25.8 | 36.6 | .824 |
| Females | -12.3 | 12.0 | -36.4 | 11.1 | .302 |
| Exposed * Females | 11.54 | 18.9 | -28.0 | 48.9 | .537 |

^Control group and males set as reference. Mixed model adjusted for age, area, education, occupation, BMI, household size; SE = standard error; CI = confidence interval^
